# Psychosocial Factors Associated with dizziness and chronic dizziness: a nationwide cross-sectional study

**DOI:** 10.1186/s12888-023-05464-7

**Published:** 2024-01-02

**Authors:** Yuna Jang, Hyun Jung Hur, Bumhee Park, Hye Youn Park

**Affiliations:** 1 Department of Psychiatry, Seoul National University Bundang Hospital, Seongnam, South Korea; 2 Department of Biomedical Informatics, Ajou University School of Medicine, Suwon, Republic of Korea; 3 Office of Biostatistics, Medical Research Collaborating Center, Ajou Research Institute for innovative Medicine, Ajou University Medical Center, Suwon, Republic of Korea; 4 Department of Psychiatry, Seoul National University College of Medicine, Seoul, South Korea

**Keywords:** Dizziness, Sex, Stress, Depression, Sleep

## Abstract

**Background:**

Dizziness is a common symptom in adults, and chronic dizziness, such as persistent postural-perceptual dizziness, is also frequently reported and affects the quality of life of patients. This study aimed to identify psychosocial factors related to dizziness and chronic dizziness in a large-scale nationwide cohort.

**Methods:**

This population-based cross-sectional study used the database of the Eighth Korea National Health and Nutrition Examination Survey in 2020. Data from 4,147 adults over 40 years old were analyzed, and 1,102 adults who experienced dizziness were included in the dizziness cohort. Demographic data, medical conditions, comorbidities, functional status variables, nutritional variables and psychological variables were collected. The pattern of depressive symptoms according to the severity of dizziness was analyzed by network analysis.

**Results:**

The prevalence rate of dizziness was 24.6% in the general population, and chronic dizziness (≥ 3 months) developed in 210 of 1,102 (17.1%) individuals who experienced dizziness. Multiple logistic regression analysis revealed that female sex, stress, and depression were associated with dizziness. Chronic dizziness was related to tympanic abnormalities, diabetes, short sleep duration, and higher levels of stress and depression. Psychomotor retardation/agitation was a central symptom of depression in patients with chronic dizziness.

**Conclusions:**

This study found sex differences in factors associated with dizziness and identified psychosocial factors linked to chronic dizziness. Focusing on somatic factors rather than depressive symptoms may benefit patients with chronic dizziness.

**Supplementary Information:**

The online version contains supplementary material available at 10.1186/s12888-023-05464-7.

## Background

The lifetime prevalence of dizziness is 16.9–23.2% [1], and 10.9–59.2% of those who reported dizziness experienced it in the past year [2]. Several studies have shown that dizziness causes poor quality of life and interrupts daily activities [3, 4]. Dizziness increases social costs by decreasing job efficiency and increasing turnover and resignations [5]. In particular, chronic dizziness is frequently reported in patients with dizziness, and 15–20% of patients who visit the dizziness center report phobic postural vertigo (PPV) or chronic subjective dizziness (CSD) [6]. Staab et al. initially proposed CSD, which includes persistent non-vertiginous dizziness over 3 months, subjective imbalance, and motion-hypersensitivity without oto-neurological abnormalities [7]. Then, the concept and diagnostic criteria of persistent postural-perceptual dizziness (PPPD) were released and PPPD was integrated and replaced CSD and PPV [8]. PPPD has become one of the most common causes of chronic dizziness in adults [9].

Previous studies have shown that dizziness is affected by a variety of factors, with sex, age, and underlying diseases, such as stroke, as known risk factors for dizziness [10, 11]. It has also been suggested that health behaviors such as sleep duration and physical activity, as well as nutritional imbalance, can have a complex effect on dizziness [12, 13]. Psychosocial factors are also associated with dizziness and chronic dizziness. Previous studies have reported that chronic dizziness is partially related to ‘psychogenic’ causes, and may also cause secondary anxiety or depression [14, 15]. Mental disorders, including anxiety disorders, frequently co-occur with balance problems [8, 16]. Additionally, anxiety or neurotic personality traits are more likely to predispose patients to PPPD [9]. Considering that anxiety interferes with postural control and spatial orientation, the psychosocial factors involved in the mechanism and epidemiology of dizziness and its chronicity should be investigated [17]. Furthermore, it is necessary to examine whether psychosocial factors are influential even when considering various factors that affect dizziness.

We hypothesized that psychosocial factors are related to dizziness. In pursuit of this objective, we aim to explore the factors associated with dizziness by comparing individuals who have experienced dizziness with those who have not. Our ultimate goal is to ascertain whether psychosocial factors retain their significance in influencing dizziness, even when these variables are controlled for. Furthermore, in this study, our aim is to comprehensively elucidate the elements associated with the chronicity of dizziness within a large-scale nationwide cohort by examining the distinct factors influencing chronic dizziness. Previous research has predominantly focused on comparing individuals with dizziness to those without [10], as well as on comparing chronic dizziness patients to non-dizziness control groups [18]. Therefore, we investigate the factors that influence the development of chronic dizziness among individuals who have experienced dizziness, with the aim of exploring the key contributors to the chronicity of dizziness. Additionally, this study aims to present therapeutic suggestions by identifying the patterns of psychosocial factors according to the severity of dizziness using network analysis.

## Methods

### Study design and participants

This cross-sectional study used data from the Eighth Korea National Health and Nutrition Examination Survey (KNHANES VIII-2) by the Korea Centers for Disease Control and Prevention in 2020. KNHANES utilized a complex sampling design (two-stage stratified cluster sampling) to extract the population. A total of 192 districts were selected for the KNHANES, and 25 households were sampled from each district using stratified cluster sampling. Health behaviors, the prevalence of chronic diseases, food intake status, and nutritional status of Koreans are assessed every year by this survey, and the sample weights are provided with consideration of non-response rates and post-stratification. Thus, by applying the sample weights, the data analysis represents the entire Korean population. This survey was conducted after approval by the Institutional Review Board of the Korea Centers for Disease Control and Prevention (2018-01-03-2C-A). Written informed consent was obtained from all participants before the survey, and this study protocol was approved by the Institutional Review Board of Seoul National University Bundang Hospital (X-2303-818-901).

The Eighth KNHANES (2020) included 3,314 households and 7,359 participants. Of these, the otolaryngologic examination, which included questions about dizziness, was performed only for those aged 40 years and older. Therefore, 4,147 adults over 40 years of age were included in the entire cohort, and 1,102 adults who reported experiencing dizziness were included in the dizziness cohort (Supplementary Fig. S1).

### Definition of dizziness

Adults over 40 years of age who responded that they had experienced dizziness or abnormal balance within the last 12 months were included in the dizziness group. Among those who reported dizziness within the last 12 months, individuals who answered “Yes” to any of the following questions were specified as the chronic dizziness group: “Have you felt uncomfortable maintaining your posture while standing for more than the last 3 months?”; “Have you felt uncomfortable walking for more than the last 3 months?”; “Have you felt chronic dizziness for more than the last 3 months?”; “Have you fallen repeatedly for more than the last 3 months?”. Participants categorized as episodic dizziness group were individuals from the dizziness group who answered “No” to all questions related to chronic dizziness, indicating that they had experienced dizziness for less than 3 months.

### Detecting the associated factors

The KNHANES includes self-report questionnaires regarding participants’ health and nutritional status. Certified inspectors conducted blood and urine sample testing and an otolaryngological examination. Sex (male and female), age (40s, 50s, 60s, 70s, 80s, or older), household income (lower, lower-middle, upper-middle, and upper level), and education (elementary school or below, middle school, high school, or university or above) were included as demographic variables. The prevalence of a tympanic abnormality was included as an otolaryngological examination variable, and the impedance audiometry results were classified as a tympanic abnormality if they were type B, C, or flat tympanogram. Waist circumference (cutoff: 90 cm for males and 80 cm for females), serum triglyceride (TG) level (increased serum TG level: ≥ 150 mg/dL), and serum high-density lipoprotein (HDL) cholesterol level (low serum HDL level: ≤ 40 mg/dL for males and ≤ 50 mg/dL for females) were used as the physical examination variables. Diagnoses of hypertension and diabetes, as well as a history of stroke, myocardial infarction, and arthritis were the comorbidity variables. The health behavior variables included alcohol consumption (Yes: more than once per month), smoking (current, former, or never smoker), and sleep duration (≤ 5, 6, 7, 8, or ≥ 9 h per day). The functional status variables were limited functional status due to visual acuity (no limitation, limitation due to visual acuity, limitation but not due to visual acuity) and activities of daily living (ADL; no difficulty with ADL, partial difficulty with ADL, cannot perform ADL). Stress, anxiety, and depression were included as psychological variables. The answer to the question, “How much stress do you usually feel in your daily life?” was used for stress perception (very much, much, little, none), and for anxiety, the responses to the degree of depression and anxiety among the EuroQol-5D (EQ-5D) questions (very much, much, none), and the Patient Health Questionnaire-9 (PHQ-9) total score were used for depression. The PHQ-9 is a self-report scale about the cognitive/affective domain (“little interest”, “depressed mood”, “guilty feeling”, “concentration problems”, “suicidal ideation”) and the somatic domain (“reduced/increased sleep”, “feeling tired”, “reduced/increased appetite”, “psychomotor retardation/agitation”). In this study, the internal consistency of the PHQ-9 was 0.80.

Only nutritional variables were included in the nutritional model with sex and age. The nutritional variables were daily intake of water, food, carbohydrates, protein, fat, dietary fiber, calcium, phosphorus, iron, sodium, potassium, beta-carotene, vitamin A, vitamin B1, vitamin B2, vitamin B3, vitamin C, vitamin D, and vitamin E, calcium, phosphorus, iron, vitamin A, vitamin B1, vitamin B2, vitamin B3, and vitamin C intake through dietary supplements.

### Statistical analysis

All analyses were conducted using complex sampling analysis to account for elements of complex sampling design, including strata, clusters, and weight. Differences in the demographic data were detected with the *t*-test for continuous variables and the χ2 test for categorical variables. Multiple logistic regression analysis was used to identify the variables related to dizziness among the entire cohort. In addition, subgroup analyses were conducted for dizziness based on sex. Chronic dizziness was used in the dizziness cohort as a dependent variable in the multiple logistic regression analysis. Only variables that were significant in the univariable logistic regression analysis were included in the multiple logistic regression analysis. The nutritional model was excluded because it was not significant. The odds ratios and 95% confidence intervals (CI) were calculated, and a two-sided p-value < 0.05 was considered significant. No multicollinearity was detected in the model (variance inflation factor < 2.0), and all analyses were performed with SPSS ver. 23.0 software (IBM Corp., Armonk, NY, USA).

Additionally, network analysis was conducted to confirm the pattern of depression according to dizziness severity. The network included 9 nodes of the PHQ-9 items that measure depression. Edges indicated statistical dependency between nodes, and thicker edges indicated a larger absolute scale and weight. The extended Bayesian information criterion Glasso model was used to create the network [19]. The R package qgraph (v.1.6.9) was used to visualize the network. Strength, betweenness, and closeness were calculated as centrality indices. Correlation stability coefficients (CS-coefficients), an indicator of the stability of centrality indices, were calculated using the R package bootnet (v.1.5), and the case-dropping subset bootstrap results for the stability of centrality are shown in Supplementary Fig.S2. As a result, strength was the most reliable, and since it was the only CS-coefficient value > 0.25 [20] in the episodic dizziness and chronic dizziness groups, only strength was interpreted in this study. The strength of a node indicated the sum of all connections to adjacent nodes, and a higher strength means that it was strongly linked to the connected node. Finally, edge-weight accuracy was estimated with the non-parametric bootstrap method with 10,000 bootstrap repetitions (Supplementary Fig.S3). This approach provided information about whether certain edges are significantly different from other edges.

## Results

### Prevalence of dizziness

Of the 4,147 participants, 24.6% (SE: 0.9%) reported experiencing dizziness or abnormalities in balance within the last 12 months. In the entire cohort, there were differences in age, sex, household income, education level, serum TG level, hypertension diagnosis, arthritis diagnosis, smoking and alcohol consumption, sleep duration, a limited functional status due to visual acuity, ADL, stress, anxiety, and all areas of depression (Table 1). Of the 1,102 participants who experienced dizziness within the last 12 months, 17.1% (SE: 1.6%) experienced chronic dizziness for more than 3 months. Differences were detected between the chronic dizziness and dizziness groups for age, sex, household income, tympanic abnormalities, hypertension, diabetes, arthritis diagnoses, sleep duration, ADL, stress level, and all areas of depression (Table 1).

**Table 1:** Demographics of dizziness vs. non-dizziness and chronic dizziness vs. non-chronic dizziness groups

	Entire cohort (n = 4,147 ^c^)			Dizziness (n = 1,102 ^c^)		
	Weighted frequency percent (SE) / Weighted means (SE)			Weighted frequency percent (SE) / Weighted means (SE)		
	Dizziness (n = 1,102 ^c^)	Non-dizziness (n = 3,045 ^c^)	*p*	Chronic dizziness (n = 210 ^c^)	Episodic dizziness (n = 892 ^c^)	*p*
**Demographic data**						
Age ^b^			< .001			.043
40–49	24.5 (1.9)	29.8 (1.4)		18.6 (3.8)	25.7 (2.1)	
50–59	27.3 (1.9)	31.2 (1.2)		23.5 (4.6)	28.1 (2.0)	
60–69	21.5 (1.4)	22.3 (1.1)		20.7 (3.9)	21.6 (1.4)	
70–79	17.7 (1.3)	12.1 (0.8)		22.3 (3.3)	16.7 (1.3)	
≥ 80	9.1 (1.0)	4.7 (0.5)		14.8 (2.5)	7.9 (1.1)	
Sex ^b^			< .001			.034
Male	36.9 (1.5)	51.4 (0.9)		29.2 (3.5)	38.5 (1.8)	
Female	63.1 (1.5)	48.6 (0.9)		70.8 (3.5)	61.5 (1.8)	
Household income ^b^			< .001			.027
Lower	26.2 (2.1)	16.0 (1.2)		35.9 (4.0)	24.1 (2.4)	
Lower-middle	24.5 (1.7)	22.4 (1.3)		27.8 (4.3)	23.8 (1.7)	
Upper-middle	25.3 (1.9)	29.2 (1.3)		14.9 (3.9)	27.5 (2.1)	
Upper	24.1 (2.0)	32.4 (1.9)		21.4 (5.3)	24.6 (2.0)	
Education ^b^			< .001			.146
Elementary school and below	25.0 (1.9)	15.2 (1.0)		32.4 (4.9)	23.7 (2.0)	
Middle school	12.4 (1.1)	10.7 (0.7)		14.3 (2.9)	12.0 (1.3)	
High school	31.8 (1.8)	36.3 (1.4)		30.5 (4.3)	32.1 (2.0)	
University or above	30.8 (2.3)	37.7 (1.9)		22.8 (5.0)	32.2 (2.4)	
**Otologic examination**						
Tympanic abnormality ^b^	6.7 (0.8)	6.4 (0.5)	.741	10.1 (2.3)	6.0 (0.8)	.037
**Physical examination**						
waist circumference ^b^			.346			.668
<cut off	45.9 (1.9)	47.9 (1.1)		47.5 (4.3)	45.5 (2.0)	
≥cut off	54.1 (1.9)	52.1 (1.1)		52.5 (4.3)	54.5 (2.0)	
Serum TG level ^b^			.026			.340
<150mg/dL	71.5 (1.9)	66.6 (1.1)		75.0 (4.0)	70.8 (2.0)	
≥150mg/dL	28.5 (1.9)	33.4 (1.1)		25.0 (4.0)	29.2 (2.0)	
Serum HDL level ^b^			.230			.616
>cut off	62.5 (1.8)	64.9 (1.2)		60.6 (4.3)	62.9 (2.0)	
≤cut off	37.5 (1.8)	35.1 (1.2)		39.4 (4.3)	37.1 (2.0)	
**Comorbidity**						
Hypertension ^b^	34.5 (1.7)	29.8 (1.0)	.007	43.5 (3.7)	32.6 (1.9)	.008
Diabetes ^b^	13.2 (1.3)	13.7 (0.7)	.720	22.3 (3.3)	11.3 (1.2)	< .001
Stroke ^b^	3.4 (0.6)	2.2 (0.3)	.074	4.7 (1.7)	3.1 (0.6)	.289
Myocardial infarction ^b^	4.3 (0.6)	3.3 (0.4)	.142	5.9 (1.6)	4.0 (0.6)	.224
Arthritis ^b^	22.1 (1.5)	11.7 (0.6)	< .001	32.8 (3.7)	20.2 (1.7)	.002
**Health behavior**						
Smoking ^b^			< .001			.349
Never smoker	62.9 (1.8)	55.4 (1.0)		68.2 (3.5)	61.8 (2.0)	
Former	22.9 (1.3)	26.6 (0.9)		18.9 (3.1)	23.8 (1.4)	
Current	14.2 (1.3)	17.9 (1.0)		12.9 (3.0)	14.5 (1.5)	
Alcohol consumption, more than once per month ^b^	48.5 (2.0)	57.7 (1.2)	< .001	43.6 (4.9)	49.5 (2.1)	.269
Sleep hours (4,139) ^b^			.002			< .001
≤5 hr	23.9 (1.4)	18.0 (0.7)		31.6 (3.5)	22.3 (1.5)	
≤ 6 hr	22.3 (1.4)	25.7 (1.0)		13.8 (2.4)	24.0 (1.5)	
≤ 7 hr	23.8 (1.6)	27.8 (1.1)		14.0 (3.1)	25.8 (1.7)	
≤8 hr	17.2 (1.4)	16.4 (0.8)		15.7 (3.7)	17.5 (1.6)	
≥9 hr	12.9 (1.4)	12.2 (0.8)		25.0 (3.8)	10.5 (1.3)	
**Functional status**						
Limited functional status due to visual acuity ^b^			< .001			.084
No limitation	87.7 (1.0)	94.3 (0.5)		81.9 (3.1)	88.7 (1.1)	
Limitation due to visual acuity	0.5 (0.2)	0.3 (0.1)		1.0 (1.0)	0.4 (0.2)	
Limitation but no due to visual acuity	11.8 (1.0)	5.4 (0.4)		17.1 (2.8)	10.8 (1.1)	
Activity of daily living ^b^			< .001			.008
No difficulty with ADL	88.4 (1.1)	95.6 (0.5)		80.7 (3.3)	89.7 (1.2)	
Partial difficulty with ADL	10.9 (1.0)	4.2 (0.5)		17.6 (3.1)	9.7 (1.1)	
Cannot perform ADL	0.7 (0.3)	0.2 (0.1)		1.7 (1.0)	0.6 (0.3)	
**Psychological variable**						
Stress ^b^			< .001			.003
None	13.1 (1.2)	16.7 (0.9)		9.4 (1.9)	13.9 (1.4)	
Little	55.7 (1.5)	60.8 (1.0)		47.0 (3.4)	57.5 (1.8)	
Much	26.5 (1.6)	18.5 (1.0)		36.5 (3.9)	24.4 (1.8)	
Very much	4.7 (0.7)	3.9 (0.5)		7.1 (2.0)	4.2 (0.7)	
Anxiety/Depression ^b^			< .001			.160
None	84.0 (1.4)	93.2 (0.6)		81.0 (3.4)	84.5 (1.5)	
Much	15.0 (1.3)	6.5 (0.6)		16.7 (3.3)	14.8 (1.5)	
Very much	1.0 (0.3)	0.4 (0.1)		2.4 (1.2)	0.7 (0.3)	
Depression (PHQ-9 total score) ^a^	3.11 (0.16)	1.79 (0.08)	< .001	5.02 (0.04)	2.79 (0.16)	< .001
Cognitive/affective domain ^a,d^	0.24 (0.02)	0.13 (0.01)	< .001	0.37 (0.05)	0.22 (0.02)	.004
Somatic domain ^a,d^	0.48 (0.02)	0.29 (0.01)	< .001	0.80 (0.06)	0.43 (0.02)	< .001

^a^ Data given as weighted mean (weighted standard error)

^b^ Data given as weighted frequency percent (weighted standard error)

^c^ Non-weighted frequency

^d^ Subtype of PHQ-9.

SE, standard error; TG, triglycerides; HDL, high-density lipoprotein; ADL, activity of daily living; PHQ-9, Patient Health Questionnaire-9

### Factors associated with dizziness

Significant variables in the univariable logistic regression analysis are shown in Table 2. Sex, stress perception, and depression were significantly related to dizziness in multiple logistic regression analyses. Women were more likely to experience dizziness than men, and experiencing some level of stress increased the probability of developing dizziness compared to no stress. In addition, high depression scores were associated with dizziness. In the subgroup analysis by sex, arthritis diagnosis, smoking, high-stress level, and high depression level were associated with dizziness in females, whereas old age, low education level, non-drinking, high-stress level, and high depression level were associated with dizziness in males (Table 3).

**Table 2:** Logistic regression analysis results for dizziness in entire cohort (n = 4,147)

	Univariable analysis		Multivariable analysis	
	OR (95% CI)	*p*	OR (95% CI)	*p*
**Demographic data**				
Age				
40–49	1		1	
50–59	1.07 (0.83–1.38)	0.612	1.06 (0.79–1.43)	0.685
60–69	1.17 (0.92–1.49)	0.186	0.98 (0.74–1.31)	0.890
70–79	1.79 (1.40–2.28)	< 0.001	1.29 (0.84–1.99)	0.235
≥ 80	2.36 (1.71–3.25)	< 0.001	1.43 (0.77–2.64)	0.257
Sex				
Male	1		1	
Female	1.81 (1.54–2.13)	< 0.001	1.79 (1.34–2.39)	< 0.001
Household income				
Lower	2.21 (1.73–2.81)	< 0.001	1.09 (0.75–1.58)	0.666
Lower-middle	1.47 (1.20–1.81)	< 0.001	1.05 (0.81–1.36)	0.726
Upper-middle	1.17 (0.95–1.44)	0.133	0.95 (0.77–1.18)	0.667
Upper	1		1	
Education				
Elementary school and below	2.01 (1.63–2.48)	< 0.001	1.24 (0.86–1.79)	0.239
Middle school	1.41 (1.10–1.81)	0.007	1.2 (0.83–1.74)	0.333
High school	1.07 (0.86–1.34)	0.522	1 (0.77–1.29)	0.973
University or above	1		1	
**Otologic examination**				
Tympanic abnormality	1.05 (0.77–1.43)	0.741	-	-
**Physical examination**				
waist circumference				
<cut off	1		-	-
≥cut off	1.08 (0.92–1.28)	0.346	-	-
Serum TG level				
<150 mg/dL	1		1	
≥150 mg/dL	0.79 (0.65–0.97)	0.026	0.92 (0.73–1.15)	0.443
Serum HDL level				
>cut off	1		-	-
≤cut off	1.11 (0.94–1.32)	0.230	-	-
**Comorbidity**				
Hypertension	1.24 (1.06–1.44)	0.007	1 (0.81–1.23)	0.999
Diabetes	0.96 (0.75–1.22)	0.720	-	-
Stroke	1.54 (0.95–2.49)	0.077	-	-
Myocardial infarction	1.32 (0.91–1.91)	0.144	-	-
Arthritis	2.14 (1.75–2.62)	< 0.001	1.34 (0.98–1.82)	0.065
**Health behavior**				
Smoking ^b^				
Never smoker	1		1	
Former	0.76 (0.63–0.91)	0.003	1.33 (0.98–1.81)	0.063
Current	0.70 (0.55–0.89)	0.004	1.24 (0.86–1.79)	0.250
Alcohol consumption, more than once per month	0.69 (0.57–0.83)	< 0.001	0.85 (0.69–1.04)	0.118
Sleep hours				
≤5 h	1.55 (1.23–1.96)	< 0.001	1.05 (0.78–1.42)	0.733
≤ 6 h	1.01 (0.80–1.29)	0.918	0.97 (0.73–1.27)	0.797
≤ 7 h	1		1	
≤8 h	1.23 (0.93–1.62)	0.145	1.29 (0.94–1.75)	0.109
≥9 h	1.23 (0.92–1.66)	0.165	0.75 (0.45–1.28)	0.290
**Functional status**				
Limited functional status due to visual acuity				
No limitation	1		1	
Limitation due to visual acuity	1.73 (0.51–5.83)	0.373	0.54 (0.13–2.3)	0.401
Limitation but no due to visual acuity	2.34 (1.87–2.93)	< 0.001	1.19 (0.83–1.69)	0.345
Activity of daily living				
No difficulties in ADL	1		1	
Partial difficulties in ADL	2.81 (2.11–3.75)	0.030	1.29 (0.81–2.03)	0.278
Cannot do ADL	3.51 (1.13–10.89)	< 0.001	1.76 (0.34–9.21)	0.501
**Psychological variable**				
Stress				
None	1		1	
Little	1.17 (0.93–1.46)	0.173	1.48 (1.09–2.01)	0.014
Much	1.83 (1.40–2.39)	< 0.001	1.99 (1.4–2.85)	< 0.001
Very much	1.53 (1.01–2.31)	0.044	1.14 (0.69–1.9)	0.599
Anxiety/Depression				
None	1		1	
Much	2.58 (1.97–3.37)	<,001	1.26 (0.88–1.81)	0.209
Very much	3.00 (1.16–7.76)	0.024	0.74 (0.19–2.92)	0.660
Depression (PHQ-9 total score)	1.11 (1.08–1.14)	< 0.001	1.08 (1.04–1.12)	< 0.001

OR = odds ratio, CI = confidence interval

TG, triglycerides (increased serum TG level: ≥150 mg = dL); HDL, high-density lipoprotein (low serum HDL level: ≤40 mg/dL for male and ≤ 50 mg/dL for female); ADL, activity of daily living; PHQ-9, Patient Health Questionnaire-9; waist circumference cutoff: 90 cm for male and 80 cm for female

Note: Variables with P-value < 0.05 in univariable analysis were included in the multivariable analysis.

**Table 3:** Subgroup analysis by sex in dizziness

	Male		Female	
	OR (95% CI)	*p*	OR (95% CI)	*p*
**Demographic data**				
Age				
40–49	1		1	
50–59	0.96 (0.62–1.49)	0.870	1.18 (0.8–1.74)	0.392
60–69	0.96 (0.59–1.56)	0.877	0.94 (0.63–1.41)	0.781
70–79	2.04 (1.14–3.63)	0.016	0.67 (0.37–1.23)	0.197
≥ 80	2.29 (1.03–5.06)	0.041	0.74 (0.28–1.95)	0.542
Household income				
Lower	1.13 (0.67–1.91)	0.655	0.98 (0.59–1.62)	0.930
Lower-middle	0.92 (0.59–1.45)	0.731	1.11 (0.77–1.6)	0.579
Upper-middle	0.93 (0.63–1.35)	0.687	0.96 (0.7–1.32)	0.818
Upper	1		1	
Education				
Elementary school and below	1.28 (0.75–2.18)	0.368	1.39 (0.8–2.4)	0.239
Middle school	1.83 (1.05–3.17)	0.032	0.82 (0.52–1.29)	0.380
High school	1.32 (0.93–1.87)	0.124	0.75 (0.53–1.07)	0.110
University or above	1		1	
**Physical examination**				
Serum TG level				
<150 mg/dL	1		1	
≥150 mg/dL	1.01 (0.74–1.4)	0.929	0.87 (0.62–1.23)	0.430
**Comorbidity**				
Hypertension	0.92 (0.66–1.28)	0.615	1.11 (0.83–1.5)	0.474
Arthritis	1.27 (0.69–2.35)	0.436	1.59 (1.12–2.27)	0.010
**Health behavior**				
Smoking				
Never smoker	1		1	
Former	1.25 (0.81–1.92)	0.317	1.15 (0.68–1.93)	0.601
Current	1.09 (0.66–1.81)	0.727	1.81 (1.03–3.17)	0.040
Alcohol consumption, more than once per month	0.71 (0.51–0.99)	0.042	0.95 (0.73–1.25)	0.738
Sleep hours				
≤5 h	0.82 (0.53–1.28)	0.392	1.32 (0.94–1.85)	0.113
≤ 6 h	0.89 (0.56–1.41)	0.610	1.04 (0.74–1.48)	0.815
≤ 7 h	1		1	
≤8 h	1.09 (0.67–1.78)	0.725	1.4 (0.95–2.07)	0.088
≥9 h	0.72 (0.34–1.53)	0.395	0.68 (0.32–1.41)	0.295
**Functional status**				
Limited functional status due to visual acuity				
No limitation	1		1	
Limitation due to visual acuity	0.49 (0.05–5.05)	0.551	0.38 (0.06–2.45)	0.308
Limitation but no due to visual acuity	1.29 (0.75–2.21)	0.352	1.12 (0.66–1.88)	0.677
Activity of daily living				
No difficulties in ADL	1		1	
Partial difficulties in ADL	1.73 (0.92–3.25)	0.088	0.88 (0.49–1.56)	0.658
Cannot do ADL	1.87 (0.16–21.16)	0.613	0.76 (0.08–7.55)	0.811
**Psychological variable**				
Stress				
None	1		1	
Little	1.39 (0.84–2.32)	0.201	1.59 (1.06–2.39)	0.027
Much	2.19 (1.24–3.87)	0.007	1.97 (1.25–3.09)	0.003
Very much	1.51 (0.63–3.61)	0.351	0.97 (0.5–1.89)	0.924
Anxiety/Depression				
None	1			
Much	1.29 (0.74–2.23)	0.364	1.31 (0.86–1.97)	0.205
Very much	0.37 (0.03–5.32)	0.464	1.49 (0.32-7)	0.612
Depression (PHQ-9 total score)	1.1 (1.04–1.16)	0.001	1.07 (1.02–1.12)	0.004

OR = odds ratio, CI = confidence interval

TG, triglycerides (increased serum TG level: ≥150 mg = dL); ADL, activity of daily living; PHQ-9, Patient Health Questionnaire-9;

### Factors associated with chronic dizziness

The results of the logistic regression analysis for chronic dizziness are shown in Table 4. Among the participants who experienced dizziness, chronic dizziness was associated with a tympanic abnormality, diabetes, sleep duration, stress perception, and depression level in multiple logistic regression analyses. Individuals who had a tympanic abnormality and slept less than 5 h were more likely to have chronic dizziness. In addition, diabetes, high depression scores, and high-stress levels were associated with the probability of developing chronic dizziness.

**Table 4:** Logistic regression analysis results for chronic dizziness among individuals with dizziness experience (n = 1,102)

	Univariable analysis		Multivariable analysis	
	OR (95% CI)	*p*	OR (95% CI)	*p*
**Demographic data**				
Age				
40–49	1		1	
50–59	1.15 (0.60–2.21)	0.673	1.19 (0.58–2.42)	0.638
60–69	1.32 (0.68–2.57)	0.409	1.14 (0.54–2.4)	0.727
70–79	1.84 (1.00-3.39)	0.050	1.45 (0.65–3.25)	0.364
≥ 80	2.58 (1.35–4.94)	0.004	1.55 (0.61–3.9)	0.353
Sex				
Male	1		1	
Female	1.51 (1.03–2.22)	0.034	1.28 (0.83–1.99)	0.262
Household income				
Lower	1.72 (0.89–3.31)	0.107	-	-
Lower-middle	1.35 (0.68–2.68)	0.396	-	-
Upper-middle	0.63 (0.27–1.47)	0.279	-	-
Upper	1		-	-
Education				
Elementary school and below	1.94 (1.05–3.58)	0.035	1.01 (0.46–2.21)	0.989
Middle school	1.68 (0.80–3.55)	0.172	1.01 (0.44–2.3)	0.983
High school	1.35 (0.71–2.56)	0.360	0.97 (0.49–1.91)	0.925
University or above	1		1	
**Otologic examination**				
Tympanic abnormality	1.76 (1.03-3.00)	0.038	2.08 (1.07–4.04)	0.030
**Physical examination**				
waist circumference				
<cut off	1		-	-
≥cut off	0.93 (0.65–1.32)	0.668	-	-
Serum TG level				
<150 mg/dL	1		-	-
≥150 mg/dL	0.81 (0.52–1.25)	0.340	-	-
Serum HDL level				
>cut off	1		-	-
≤cut off	1.10 (0.75–1.63)	0.616	-	-
**Comorbidity**				
Hypertension	1.59 (1.13–2.24)	0.008	1.19 (0.76–1.87)	0.440
Diabetes	2.25 (1.49–3.41)	< 0.001	2.4 (1.44–4.02)	0.001
Stroke	1.51 (0.70–3.27)	0.292	-	-
Myocardial infarction	1.52 (0.77–2.98)	0.225	-	-
Arthritis	1.92 (1.27–2.91)	0.002	1.26 (0.76–2.11)	0.373
**Health behavior**				
Smoking				
Never smoker	1		-	-
Former	0.72 (0.46–1.12)	0.141	-	-
Current	0.81 (0.45–1.46)	0.481	-	-
Alcohol consumption, more than once per month	0.79 (0.52–1.20)	0.269	-	-
Sleep hours				
≤5 h	2.62 (1.58–4.34)	< 0.001	1.92 (1.03–3.58)	0.040
≤ 6 h	1.06 (0.58–1.91)	0.857	1.03 (0.54–1.96)	0.923
≤ 7 h	1		1	
≤8 h	1.65 (0.74–3.67)	0.221	1.6 (0.71–3.59)	0.255
≥9 h	4.40 (2.27–8.55)	< 0.001	0.74 (0.22–2.48)	0.624
**Functional status**				
Limited functional status due to visual acuity				
No limitation	1		1	
Limitation due to visual acuity	2.59 (0.2923.32)	0.394	0.94 (0.16–5.48)	0.948
Limitation but no due to visual acuity	1.71 (1.07–2.74)	0.026	0.76 (0.41–1.4)	0.375
Activity of daily living				
No difficulties in ADL	1		1	
Partial difficulties in ADL	2.01 (1.22–3.33)	0.007	1.17 (0.62–2.2)	0.621
Cannot do ADL	3.29 (0.70–5.43)	0.130	0.67 (0.09–5.04)	0.694
**Psychological variable**				
Stress				
None	1		1	
Little	1.21 (0.73-2.00)	0.465	1.41 (0.77–2.58)	0.263
Much	2.21 (1.22–4.01)	0.009	2.31 (1.14–4.68)	0.021
Very much	2.49 (1.06–5.86)	0.037	2.12 (0.7–6.48)	0.185
Anxiety/Depression				
None	1		-	-
Much	1.18 (0.71–1.96)	0.524	-	-
Very much	3.36 (0.9112.49)	0.070	-	-
Depression (PHQ-9 total score)	1.11 (1.07–1.15)	< 0.001	1.07 (1.02–1.12)	0.003

OR = odds ratio, CI = confidence interval

TG, triglycerides (increased serum TG level: ≥150 mg = dL); HDL, high-density lipoprotein (low serum HDL level: ≤40 mg/dL for male and ≤ 50 mg/dL for female); ADL, activity of daily living; PHQ-9, Patient Health Questionnaire-9; waist circumference cutoff: 90 cm for male and 80 cm for female

Note: Variables with P-value < 0.05 in univariable analysis were included in the multivariable analysis.

### Network analysis of the depression pattern based on dizziness severity

**Fig. 1:**
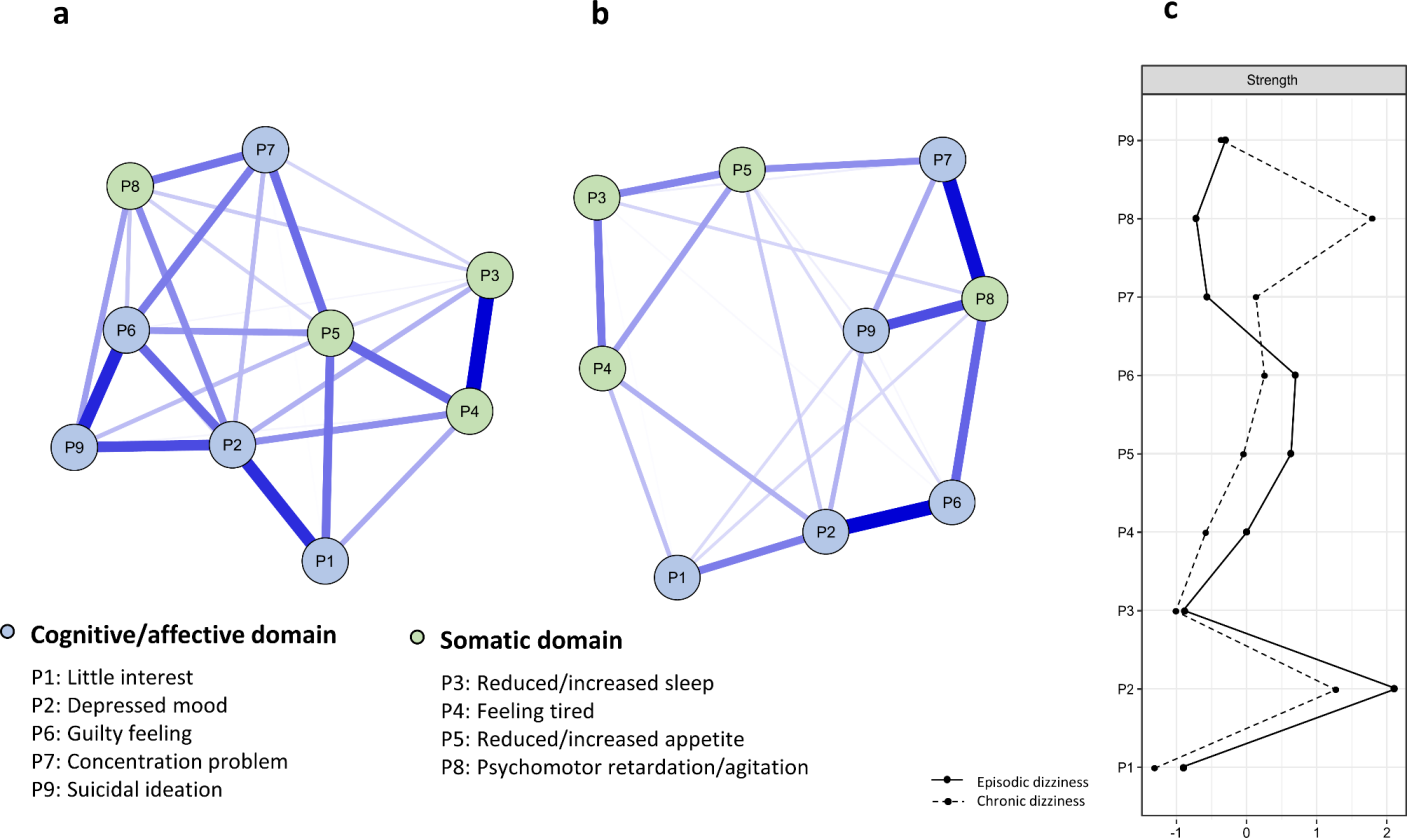
Network structure of depression and node centrality indices of strength Note: Network structure of dimension of depression in individuals with (**a**) episodic dizziness and (**b**) chronic dizziness, including (**c**) node centrality indices of strength Nodes are represented as circles, with blue nodes representing the Cognitive/Affective domain of the PHQ-9, and green nodes representing the Somatic domain of the PHQ-9. Edges are depicted as connecting blue lines, with thickness indicating the strength of association

As depression was related to episodic and chronic dizziness, a detailed network analysis was conducted. The estimated network is presented in Fig. 1. The observed differences in depression expression patterns between episodic dizziness and chronic dizziness are indicative (Fig. 1a and b). A 95% CI was estimated for each edge using the non-parametric bootstrap method (Supplementary Fig.S3). The first three edge weights were significantly stronger than the other edges. As demonstrated in Fig. 1a and b and Supplementary Fig.S3, the edge weights of P3 (reduced/increased sleep) and P4 (feeling tired), P6 (guilty feelings) and P9 (suicidal ideation), P1 (little interest) and P2 (depressed mood) were significantly stronger in the episodic dizziness group and relations of P2 (depressed mood) and P6 (guilty feelings), P7 (concentration problem) and P8 (psychomotor retardation/agitation), P8 (psychomotor retardation/agitation) and P9 (suicidal ideation) were significantly stronger in the chronic dizziness group. The nodes showing high strength in the episodic dizziness group were P2 (depressed mood), P6 (guilty feelings), and P5 (reduced/increased appetite), while P8 (psychomotor retardation/agitation), P2 (depressed mood), and P6 (guilty feelings) showed high strength in the chronic dizziness group (Fig. 1c).

## Discussion

This study investigated psychosocial factors of dizziness and chronic dizziness in a relatively large number of people using a national representative sample that reflected the general population. In our study, 24.6% of people in the general population experienced episodic dizziness. This was slightly higher than the 19.5–20.1% of respondents who said they experienced dizziness within the past year in the 2010–2012 KNHANES study in Korea. [10, 12, 21]. Among this population, 4.2% (SE: 0.4%) of individuals reported that they had experienced chronic dizziness over 3 months, which was similar to the 4.8% incidence of chronic dizziness in a previous KNHANES study [18].

This study demonstrated that sex, stress level, and depression level were related to dizziness. Dizziness was observed frequently, particularly in females, which agreed with previous studies [4, 22, 23]. The female predominance in dizziness has been partly explained by the female predominance in comorbid conditions, such as migraine [24] and postural orthostatic tachycardia syndrome [25]. These sex differences in dizziness can also be explained by differences in sex hormones. Sex hormones regulate neural effector mechanisms that affect the control of vascular tone through peripheral sympathetic and parasympathetic neural activities, as well as the central adrenergic and serotonergic systems [26]. Differences in factors affecting the experience of dizziness were detected according to sex in the subgroup analysis. Smoking and an arthritis history were related to dizziness in females. The result that smoking was associated with dizziness is consistent with previous studies [27, 28]. Smoking adversely affects the vascular supply to the inner ear, making it easier to develop dizziness [29, 30]. Arthritis was also associated with dizziness in women, which is consistent with a previous cohort study [31]. Arthritis is more prevalent in women [32], and patients with arthritis are more likely to experience postural instability [33, 34] because musculoskeletal function plays a crucial role in the balance system [35]. In addition, medications for arthritis, such as minocycline and opioids, may cause dizziness as an adverse effect [36, 37]. Older age, low education level, and alcohol consumption were associated with dizziness in males. Various studies have reported that the incidence of dizziness increases with age, which is mediated by multifactorial pathways, including cardiovascular comorbidities and vestibular dysfunction [29, 38]. Furthermore, prior studies have reported sex disparities in the diseases that induce dizziness and their manifestation patterns [39]. Specifically, there is a pronounced increase in the prevalence of cerebrovascular and degenerative diseases with age, particularly in males [40]. This corroborates our study’s findings, highlighting the association of age as a contributing factor to dizziness in males. Previous studies have suggested that a higher educational level has a protective effect on vestibular dysfunction [41, 42]. The result that people who drink alcohol more than once per month are less dizzy requires caution in interpretation. Drinking decreases perceptual function due to neuropathy when it lasts for a long time which can increase the dizziness [43]. Our study showed the opposite result because we analyzed only whether participants consumed alcohol more than once a month and did not subdivide the amount of alcohol consumed each month.

High levels of stress and depression were particularly associated with dizziness in both male and female, which is consistent with studies showing that stress and depression are closely related to dizziness [44, 45], but it is difficult to conclude that stress and depression are causes or predictors of dizziness. Our results show that depression and stress were related to dizziness, which supports the idea that depression or stress may underlie dizziness. Patients with dizziness report depression and anxiety, and patients with mood disorders often experience dizziness [46]. Previous studies have found direct associations between the vestibular nuclei and the brainstem region, including the limbic system, indicating that stimuli affecting balance control can have a significant effect on anxiety-related pathways [47]. In addition, high stress hormone levels due to stress can affect the vestibular system and cause dizziness [48].

Among those who experienced dizziness, the factors related to chronic dizziness were a tympanic abnormality, short sleep duration, diabetes, and high levels of stress and depression. Indeed, various otological disorders with tympanic abnormalities, such as chronic otitis media and perilymphatic fistula, may induce dizziness or vertigo [49]. In this study, we observed that short sleep duration (< 5 h) was associated with chronic dizziness. One study reported that an abnormal sleep pattern, such as < 5 h of sleep or > 9 h of sleep, is associated with dizziness, as an abnormal sleep pattern affects general physical health [12]. Several previous studies have suggested that sleep deprivation precedes chronic dizziness [50], and poor sleep quality exacerbates dizziness in patients with chronic dizziness [51]. Although the mechanism of chronic dizziness and sleep has not been clarified, previous findings have suggested that sleep deprivation may sensitize the vestibular system related to dizziness [52] and disrupted circadian rhythms may alter balance control [53]. Considering that the subjects in the chronic dizziness group were older than those in the episodic dizziness group, this study showed that diabetes mellitus was associated with chronic dizziness [54]. Indeed, diabetes is a predictor of imbalance and fall risk in older adults [55]. Chronic and recurrent hyperglycemia from diabetes affects the balance system via damage to the vestibular organs, and diabetic neuropathy and retinopathy increase gait instability [56].

Stress was associated with chronic dizziness. Stress interplays with central vestibular function via direct and indirect neuroendocrine pathways. Excessive stress may impair vestibular compensation and can induce dizziness [57]. A previous study reported an increase in cortisol levels in patients experiencing chronic dizziness [58]. Furthermore, depression was also strongly related to prolonged dizziness in this study. Specifically, the network analysis revealed that the domains of depression influencing the episodic and chronic dizziness groups were different. In the episodic dizziness group, a depressed mood represented by the cognitive/affective domain, and psychomotor retardation/agitation represented by the somatic domain in the chronic dizziness group showed the strongest relationship with other depressive symptoms. In particular, symptoms in the chronic dizziness group were linked to psychomotor retardation/agitation as a central symptom among various depressive symptoms. Psychomotor retardation is defined as a decrease in activity and motor skills, resulting in slowed or sluggish movements, while psychomotor agitation is characterized by increased restlessness and activity [59, 60]. These are representative somatic symptoms of depression. It has been reported that the pathophysiological mechanism of chronic dizziness is related to the processing, regulation, and psychomotor regulation of somatosensory signals rather than affective regulation [9]. Excessive self-observation regarding posture and vigilance accompanied by a high level of anxiety lead to the amplification of somatosensory information and subjective dizziness [9]. This vicious cycle, repeated by somatosensory stimuli, partially explains chronic dizziness. Our results should be interpreted cautiously because depression and stress may be predictors of dizziness, but they may also represent secondary depression due to chronic dizziness or co-morbid depression.

Our study had several limitations. First, this study used cross-sectional designed data, so causal relationships could not be concluded. Second, in this study, dizziness was assessed exclusively through self-reported surveys that inquired about the presence of dizziness over a defined period. Consequently, the potential for recall bias cannot be discounted. Furthermore, given that it was not evaluated using a standardized scale, prudent consideration is essential when interpreting these findings, particularly when discussing them in conjunction with conditions such as PPPD. Third, dizziness and vertigo were not distinguished and the various types of chronic dizziness were not distinguished. Nevertheless, this study has confirmed the tendency for dizziness in nationally representative data after considering demographic variables, blood test results, underlying diseases, and psychological factors. Notably, while previous KNHANES studies have only confirmed a history of depression, this study used the validated PHQ-9 to confirm the current state of depression and explore the specific symptom dimensions related to chronic dizziness severity using network analysis.

## Conclusions

This is the first study to analyze the relationship between chronic dizziness and psychosocial factors in a nationwide cohort. Specifically, this study investigated the factors related to dizziness and factors contributing to the chronicity of dizziness. Furthermore, this study ascertained the predominant domains within various dimensions of depression, contingent on the severity of dizziness. Sex and psychosocial factors, such as stress and depression, were associated with dizziness, and related factors were different between the sexes. These findings support the idea that there might be sex differences in the pathophysiological mechanisms of dizziness. Furthermore, psychosocial factors, including sleep, stress, and depression as well as diabetes and tympanic abnormalities were associated with chronic dizziness. Especially psychomotor retardation/agitation was a central symptom among depressive symptoms in individuals with chronic dizziness. Therefore, it would be more beneficial for individuals with chronic dizziness and comorbid psychosocial factors to focus on somatic factors, such as sleep, stress, and psychomotor function, rather than depressive symptoms. Further studies are needed to investigate the clinical usefulness of screening and management of these somatic factors in patients with dizziness. Understanding the different factors and mechanisms related to dizziness and its chronicity will improve specific treatments for dizziness and improve quality of life.

### Electronic supplementary material

Below is the link to the electronic supplementary material.

**Supplementary Material 1:** Study population and detailed figures of network analysis

## Data Availability

The datasets generated and analyzed in this study are available in the Korea National Health and Nutrition Examination Survey repository. (https://knhanes.kdca.go.kr/knhanes/sub03/sub03_02_05.do)
