# Supplementary material for: Psychosocial Factors Associated with dizziness and chronic dizziness: a nationwide cross-sectional study
Source: BMC Psychiatry. 2024 Jan 2;24:13. doi: 10.1186/s12888-023-05464-7 (PMC10762808; doi:10.1186/s12888-023-05464-7)
Supplement: Supplementary file 1 — Supplementary Material 1: Study population and detailed figures of network analysis [file 12888_2023_5464_MOESM1_ESM.docx]

**Supplementary Material**


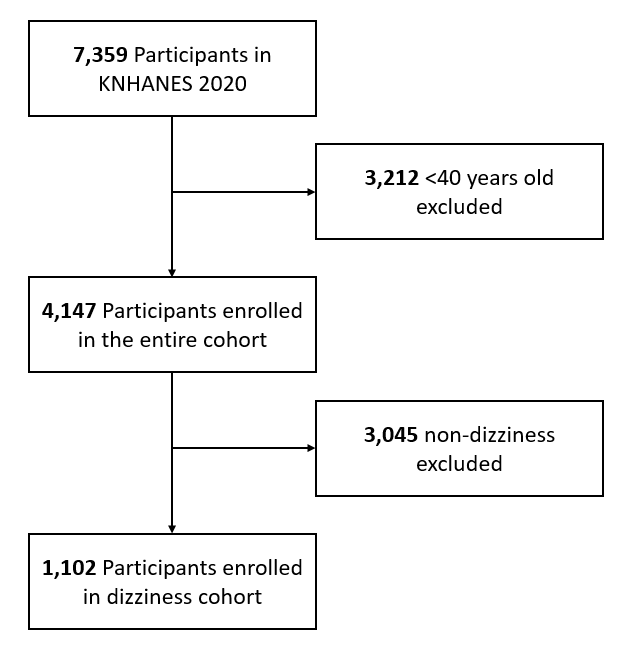


**Supplementary Fig. S1.** Study population: a flow chart of exclusions


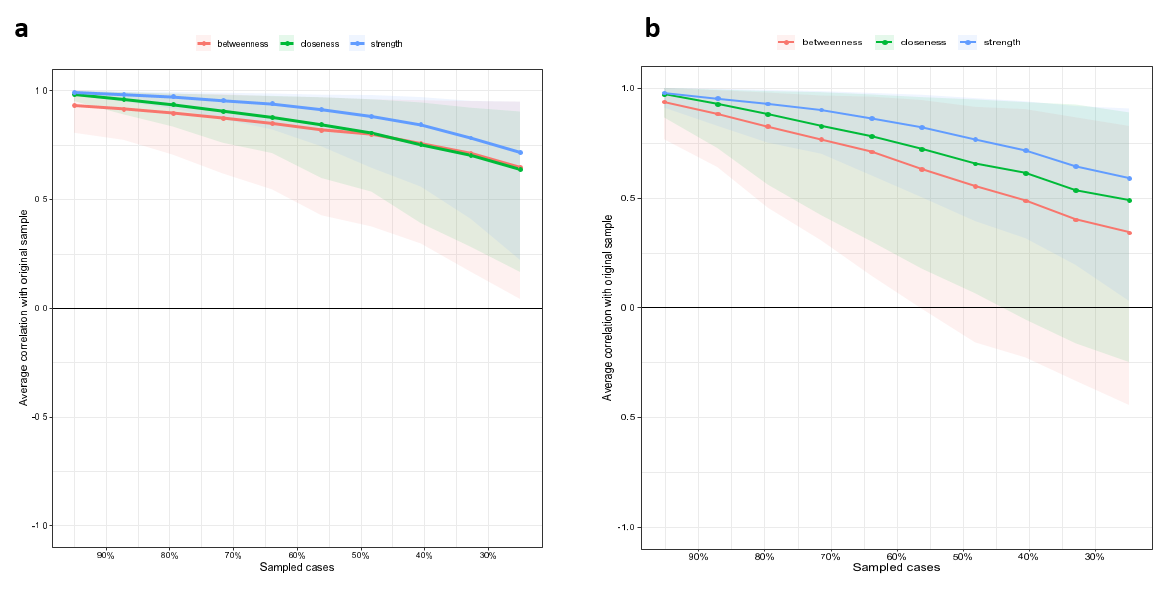


**Supplementary Fig. S2.** Stability of all centrality indices of depression dimension by the case-dropping subset bootstrap in (a) episodic dizziness and (b) chronic dizziness


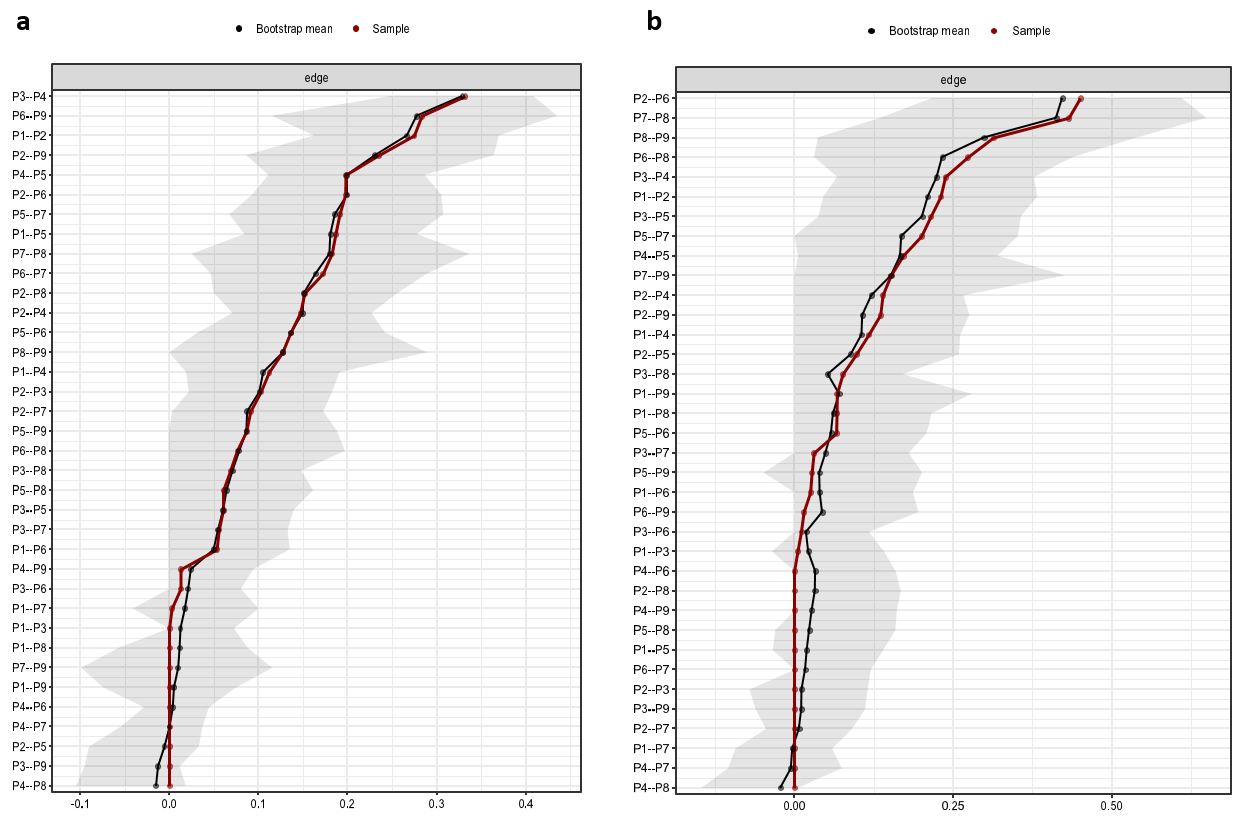


**Supplementary Fig. S3.** Bootstrapped confidence intervals for all edges of depression dimension with (a) episodic dizziness and (b) chronic dizziness
